# Features of mismatch negativity in an at-risk mental state with the traits associated with the autistic spectrum

**DOI:** 10.3389/fpsyt.2025.1620954

**Published:** 2025-08-14

**Authors:** Naohito Kaneko, Yuko Higuchi, Noa Tsujii, Shimako Nishiyama, Yukiko Akasaki, Kazuya Nagasawa, Daiki Sasabayashi, Michio Suzuki, Tsutomu Takahashi

**Affiliations:** ^1^ Department of Neuropsychiatry, Graduate School of Medicine and Pharmaceutical Sciences, University of Toyama, Toyama, Japan; ^2^ Research Center for Idling Brain Science, University of Toyama, Toyama, Japan; ^3^ Department of Child Mental Health and Development, Toyama University Hospital, Toyama, Japan; ^4^ Center for Health Care and Human Sciences, University of Toyama, Toyama, Japan; ^5^ Arisawabashi Hospital, Toyama, Japan; ^6^ Itoigawa Clinic, Niigata, Japan

**Keywords:** at-risk mental state, psychosis, event-related potential, mismatch negativity, autism spectrum disorder, autism-spectrum quotient

## Abstract

**Introduction:**

Accurately distinguishing individuals with autism spectrum disorder (ASD) from those with schizophrenia spectrum disorder (SSD) can be challenging, especially in individuals with an at-risk mental state (ARMS) for psychosis. Given the need for objective markers, we focused on mismatch negativity (MMN). This study aimed to determine whether ARMS individuals with ASD traits exhibit different MMN patterns compared to ARMS individuals without such traits and healthy controls.

**Methods:**

Forty-nine individuals with ARMS and 45 healthy controls were enrolled. The Autism-Spectrum Quotient Japanese Version (AQ-J) was used to assess ASD traits, with a cut-off of 33+ indicating high ASD traits [AQ(+)] and scores below that low ASD traits [AQ(-)]. An electroencephalogram was recorded while the participants heard standard and deviant tones in two auditory oddball paradigms: a duration-deviant (dMMN) and a frequency-deviant (fMMN). MMN amplitude and latency were analyzed at Fz and group differences were compared between patients with ARMS and healthy controls. Further, within the ARMS group, AQ(-) (*n* = 33) *vs.* AQ(+) (*n* = 16) subgroups were examined. Correlation analyses were also performed to explore the relationships between MMN measures and clinical/cognitive indices.

**Results:**

No significant differences in MMN amplitude or latency were observed between the ARMS group and healthy controls. In contrast, fMMN latency in the AQ (+) group was significantly shorter than that in the AQ(-) group. Within the entire ARMS group, fMMN latency had a significant negative correlation with total AQ-J scores, especially the Communication subscale, i.e., higher ASD traits were associated with shorter fMMN latency.

**Conclusion:**

The key finding of this study was that ARMS individuals with higher ASD traits showed a shortened fMMN latency compared to those without. Distinguishing ARMS from ASD based solely on clinical symptoms is sometimes difficult, and using an objective measurement tool such as MMN latency could help identify underlying ASD features and guide more tailored interventions.

## 1 Introduction

Several studies have described individuals with both autism spectrum disorder (ASD) and schizophrenia; the broader phenotypes of these disorders clearly overlap (1). While there is considerable variation between reports, the prevalence of schizophrenia in individuals with ASD has been reported to range from 0-34.8% (2–5), which is clearly higher than that in adult general population (0.45%; World Health Organization, 2022), and ASD in schizophrenia is between 3.6-60% (6–10). Accurate diagnosis is important because of the distinct clinical courses and intervention approaches between schizophrenia and ASD patients, but they are sometimes difficult to clearly separate due to partly overlapping clinical phenotypes, such as recurrent hallucinations in ASD (2) and similar negative symptomatology (11). By contrast, there are clear phenomenological and pathophysiological differences between the schizophrenia and ASD in the following respects: onset age (adolescence or childhood), presence/absence of anomalous self-experience and reality monitoring (12, 13), behavioral pattern (repetitive, rule-based behaviors in ASD contrast with the formal thought disorder and disorganization observed in schizophrenia) (14), forms of sensory impairment (ASD shows hyper-/hypo-reactivity to sensory input, while schizophrenia has impaired sensory gating) (15, 16). Recently, the concept of at-risk mental state (ARMS) individuals was proposed (17), who are at an increased risk of developing psychosis within a relatively short period of time (approximately 30% in 2 years) (18). Their symptoms are milder and more nonspecific than those of schizophrenia, and it is more difficult to differentiate ASD and ARMS individuals. A systematic review reported that the prevalence of ASD in ARMS ranged from 1.1% to 39.6% and that of ARMS in ASD ranged from 0% to 78.0% (19). Further, a recent survey study using the PRIME Screen-Revised, a self-reported instrument for prodromal symptoms of psychosis (20) demonstrated that substantial number of first-visit ASD outpatients had subthreshold or sporadic psychotic symptoms similar to ARMS individuals (21). These data show the difficulty of distinguishing between ASD and schizophrenia-spectrum disorders (SSD), especially in early stages for psychosis, based on clinical symptoms alone, indicating the need for objective biomarkers useful for differential diagnosis.

There are several candidate biomarkers of schizophrenia, including brain structure, function, and blood markers (22). Among these, mismatch negativity (MMN), which indexes pre-attentive sensory processing using oddball tasks (e.g., changing the duration or frequency of auditory stimuli) (23–25), has emerged as a potential biomarker for psychosis (26–28). Reduced amplitude of duration MMN (dMMN) has been reported in individuals with chronic schizophrenia and early stages of psychosis, such as first-episode schizophrenia (FES) and ARMS (29–35). More specifically, MMN amplitude has consistently been reported to be reduced in schizophrenia, with a large effect size of approximately 0.9 (36, 37). Subsequent early-intervention studies indicated that smaller baseline dMMN amplitudes in individuals with an at-risk mental state (ARMS) predicted conversion to psychosis and were associated with poorer functional outcomes (38, 39). Because the generation of dMMN is dependent on N-methyl-D-aspartate (NMDA) receptor-mediated neurotransmission—a pathway long implicated in the pathophysiology of schizophrenia, dMMN has thus been viewed as a potentially useful objective biomarker for the disorder. On the other hand, the amplitude of frequency MMN (fMMN) is reportedly reduced in chronic schizophrenia but not in FES or ARMS patients (36, 40). Regarding MMN latency, the findings in schizophrenia have been inconsistent [prolonged (41, 42), shortened (43), no change (44) or not documented].

Many MMN studies have been conducted also in patients with ASD; according to a meta-analysis of 22 studies (45), dMMN amplitude is likely to be reduced especially in children/adolescents with ASD, while its latency does not appear to change in ASD regardless of age. There were no significant differences between ASD patients and controls in fMMN amplitude/latency, but low-function ASD may be characterized by shortened fMMN latency (46). Taken together with the findings in schizophrenia, MMN may serve as a biomarker of both psychosis and ASD. However, summarizing the limitations of the previous studies, MMN studies in ASD show highly variable findings across age groups and intellectual-functioning levels. In ARMS cohorts, the most robust result is a reduction in dMMN amplitude; however, fMMN, particularly latency has received little attention. Only a report has examined MMN in ASD patients who also display ARMS traits, and most ARMS studies neither control for nor stratify neurodevelopmental factors such as ASD. Although a theoretical ASD–ARMS/psychosis continuity model posits a shared abnormality in prediction-error processing (e.g., Sterzer et al., 2018) (47), no empirical work has yet asked how the ARMS subgroup with pronounced ASD traits manifests MMN alterations. Accordingly, research that explicitly examines MMN amplitude and latency in ARMS individuals stratified by ASD traits is needed to test the proposed neurophysiological continuum between ASD features and psychosis risk.

The AQ is a simple and convenient screening tool that can be easily administered to individuals with ASD traits (48). Owing to its ease of use, it has been widely employed in clinical settings both in Japan and internationally. According to the validation study of the Japanese version (AQ-J), individuals exceeding the cutoff value accounted for approximately 90% of those with ASD, and the tool demonstrated high specificity (3%) in the general population as well as strong measurement reliability (49). Having said that, the AQ is a self-report test for ASD “trait” and has the aspect that it captures only dimensional ASD traits across the broader spectrum rather than diagnoses.

This study aimed to investigate the relationship between ASD features and MMN in individuals with ARMS and to examine whether MMN could serve as a useful biomarker for identifying individuals with ASD traits in ARMS. We predicted that individuals with ARMS who have ASD traits would show different results in MMN compared to those who do not. This study may contribute to the early detection, differential diagnosis, and development of individualized interventions for both ARMS and ASD.

## 2 Materials and methods

### 2.1 Participants

A total of 49 subjects with ARMS (19 male and 30 female; mean age ± standard deviation, 18.9 ± 4.7 years), recruited from the University of Toyama Hospital or Toyama Prefectural Mental Health Centre (46) participated in this study (50). Individuals with ARMS were identified by experienced psychiatrists using the Comprehensive Assessment of At-Risk Mental State (CAARMS) (17). Subgroups of ARMS included attenuated psychotic symptoms (APS), genetic risk and worsening syndrome (GRD) and/or short-term limited intermittent psychotic symptoms (BLIPS). Eligible subjects were confirmed to have good hearing ability and physical health, based on physical examinations and standard laboratory tests. Subjects were excluded if they had a history of substance abuse or dependence, seizures, head injury, or an estimated premorbid Intelligence Quotient (IQ) <70 based on the Japanese Adult Reading Test (51). Of the 49 ARMS, 8 received antipsychotic medication (0.12 ± 0.33 mg/day, risperidone equivalent). We also recruited 45 healthy controls (H) (23 male and 22 female participants; mean age, 22.6 ± 2.6 years) from our community, university students, and hospital staff. Participants were screened for past or current Axis I disorders based on the Structured Clinical Interview for DSM-IV (SCID) (52). Additional exclusion criteria for H (in addition to those listed above) were a history of psychiatric disorders in the participants themselves or their first-degree relatives.

The Committee on Medical Ethics of the University of Toyama approved the study protocol (no. I2013006 on February 5, 2014). Written informed consent was obtained from all participants in accordance with the Declaration of Helsinki. If the participants were under 20 years old, written consent was also obtained from a parent or legal guardian.

### 2.2 Clinical assessment

Experienced psychiatrists or psychologists evaluated clinical symptoms in individuals with ARMS using the PANSS (53). The Brief Assessment of Cognition in Schizophrenia (BACS) Japanese version (54, 55), Schizophrenia Cognition Rating Scale (SCoRS) Japanese version (56, 57) and modified Global Assessment of Functioning (mGAF) (58) were used to evaluate each participant’s cognitive and social functioning. The BACS composite score was calculated by averaging the z-scores of the six primary BACS measurements.

The Autism-Spectrum Quotient (AQ) was used to assess traits associated with ASD (48). It was translated into Japanese, standardized, and is widely used in Japan as AQ-J (AQ-Japanese version) (49). The AQ-J consists of 50 items divided into five subscales with 10 questions each. The scale assesses five areas of cognitive strengths and difficulties related to ASD traits: Communication, Social Skills, Imagination, Attention to Detail, and Attention Switching. Higher scores on each subscale suggest poor communication skills, poor social skills, poor imaginations, exceptional attention to detail, and difficulties in attention switching or strong focus on attention, respectively (48). We set a score of 33 or greater, indicating a high possibility of having ASD traits [AQ(+)], and 32 or lower as AQ(-) (49).

### 2.3 MMN recording

MMNs were recorded using an auditory oddball paradigm based on an established method performed in our institute (34, 59, 60). Briefly, Electroencephalogram (EEG) recordings were obtained using a Nihon Kohden EEG device (EEG-1250 version 07-02, Nihon Kohden Corp.) or Polymate AP1532 (TEAC Corp.) and 32-channel Electrocap (Electrocap Inc.) or 32-channel MCS cap (Medical Computer Systems Ltd.) in a wave-shielded and sound-attenuated room. Auditory stimuli were delivered binaurally through headphones while participants were seated while watching a silent cartoon to stay alert without auditory interference. Two auditory oddball paradigms were employed using duration- and frequency-deviant stimuli. For the dMMN, 1500 stimuli consisting of 90% standard tones (1,000 Hz, 50 ms) and 10% deviant tones (1,000 Hz, 100 ms) were used. For the fMMN, 1,500 stimuli consisting of 90% standard tones (1,000 Hz, 50 ms) and 10% deviant tones (1,500 Hz, 50 ms) were used. The inter-stimulus interval (ISI) was fixed at 500 ms, resulting in a stimulus-onset asynchrony (SOA) of 550 ms for standard tones (50 ms) and 600 ms for dMMN deviant tones (100 ms). Auditory parameters were delivered at a 60-dB sound pressure level a 10 ms rise/fall time. The data were collected at a sampling rate of 500 Hz. The bandwidth was set at 0.53–120 Hz with a 60 Hz notch filter. The reference electrode was located at Aav and the ground electrode was at Z. Electrode impedance was less than 10 kΩ. Auditory stimuli were presented in two consecutive blocks: dMMN (first) and fMMN (second). There was 1 min break time between the two blocks. Epochs were averaged with EPLYZER II (Kissei Comtec Co., Ltd.): 600 ms (dMMN) or 500 ms (fMMN) epochs, each including a 100 ms pre-stimulus baseline. Epochs containing voltage excursions > ± 100 μV by blink, eye-movement, and body movement were manually discarded. Artifact-free epochs were averaged separately for target and non-target. The target waveforms were subtracted from the non-target ones to yield the MMN. Each epoch was baseline-corrected by subtracting the mean voltage in the −100 to 0 ms window. The amplitude and latency of the dMMN and fMMN were used as parameters. For dMMN, the peak observed 130−250 ms after the start of the sound was used as its amplitude (zero-point to peak) and latency (0 ms to peak). For fMMN, the peak observed 60−180 ms after the start of the sound was used. For statistical analyses, only the recording at Fz, which generally has the greatest amplitude compared with the other electrodes, was used as a representative of the MMN for each individual, according to previous literature (61, 62). The detailed data are provided in **Supplementary Table 1**.

### 2.4 Statistical analysis

Statistical analyses were performed using the Statistical Package for Social Sciences version 25 (SPSS Japan Inc.) and Jamovi Software (https://www.jamovi.org). The analyses covered dMMN and fMMN parameters (amplitude and latency), the AQ-J and 5 subscales (Communication, Social Skills, Imagination, Attention to Detail, and Attention Switching) as well as the PANSS, BACS, mGAF, and SCoRS scores. We used parametric statistics because the data were normally distributed (tested using the Shapiro-Wilk test). For the MMN amplitude, the polarities were negative in all participants, and their absolute values were used in the statistical analysis. Demographic and clinical data were compared between the groups using the chi-square test or two-tailed Student’s t-test. Analysis of covariance (ANCOVA) with age as a covariate was used to assess group differences in MMN parameters (amplitude and latency), because a previous study found effects of aging on MMN parameters (63). Bonferroni correction was applied within 4 parameters (k = 4), yielding a significance threshold of p < 0.0125. Degrees of freedom for each correlation were df = 47. Analysis of variance (ANOVA) with Bonferroni correction was used to assess group differences in AQ-J and its subscales in H, AQ(-) and AQ(+) group. Pearson’s correlation coefficient with a semi-partial correlation was used to calculate the correlation between MMN parameters and clinical data, with only MMN parameters controlled by age. Because a significant correlation was found between fMMN latency and AQ-J, we also investigated the relationship between fMMN latency and AQ-J subscale scores. Bonferroni correction was applied within 5 subscales (k = 5), yielding a significance threshold of p < 0.01. Degrees of freedom for each correlation were df = 47. The significance level was set at *p* < 0.05, however, when comparing multiple variables, only those that were significant even after the *post-hoc* analysis were considered significant.

## 3 Results

### 3.1 Characteristics of study population

Demographic and clinical data of the H and ARMS groups are shown in **Table 1**. There were significant group differences in the AQ-J score, age, JART, and BACS, whereas the male/female ratio did not differ. Similarly, data of the AQ(-) and AQ(+) ARMS subgroups are shown in **Table 2**; no significant group difference was found for age, gender, JART, antipsychotic dose, percent of medication, PANSS, BACS, mGAF and SCoRS scores. The conversion ratio to psychosis did not differ between the groups. Detailed information on the AQ-J subscales is provided in **Supplementary Table 2**.

**Table 1:** Demographic and clinical data for groups H and ARMS.

	H	ARMS	Group difference^a^
	*n=*45	*n*=49	
AQ-J score	18.0 (5.7)	27.5 (7.9)	*t*_45,49_ = 6.60*, p<*0.001
Age (years)	22.6 (2.6)	18.9 (4.7)	*t*_45,49_ = -4.63, *p<*0.001
Gender (male/female)	23/22	19/30	χ² = 1.44, *p*=0.23
JART	109.0 (4.3)	99.1 (9.7)	*t*_43,48_ = -6.16, *p*<0.001
BACS^b^	0.3 (0.6)	-0.5 (0.7)	*t*_43,49_ = -5.13*, p*<0.001

All values are shown as means (standard deviations).

ARMS, at-risk mental state; AQ-J, Autism-Spectrum Quotient Japanese version; BACS, Brief Assessment of Cognition in Schizophrenia; H, healthy controls; JART, Japanese Adult Reading Test.

a Demographic differences between groups were examined by chi-square or Student’s t-test.

b BACS composite score was calculated by averaging all z-scores of the six primary measures from the BACS.

**Table 2:** Demographic and clinical data for AQ(-) and AQ(+) ARMS subgroups.

	AQ (-)	AQ (+)	Group difference^a^
	*n*=33	*n*=16	
AQ-J score	23.2 (5.7)	36.2 (3.3)	*t_33,16_* = -8.39*, p<*0.001
Age (years)	18.1 (3.7)	20.5 (6.1)	*t_33,16_* = -1.70, *p* = 0.09
Gender (male/female)	12/21	7/9	*χ²* = 0.25, *p*=0.62
JART	99.7 (8.9)	98.0 (11.3)	*t_32,16_ =* 0.57, *p*=0.58
Antipsychotic dose (mg/day, risperidone equivalent)	0.1 (0.4)	0.1 (0.3)	*t_32,16_ =* 0.57, *p*=0.67
Antipsychotic medication (yes/no) (%)	5/28 (15%)	3/13 (18%)	*χ²* = 0.062, *p*=0.80
PANSS: Total	52.0 (9.8)	55.4 (12.3)	*t_31,16_* = -1.04, *p*=0.30
PANSS: Positive	11.8 (3.4)	13.8 (4.0)	*t_31,16_* = -1.79, *p*=0.08
PANSS: Negative	13.4 (5.7)	13.4 (4.4)	*t_31,16_* = -0.01, *p*=0.99
PANSS: General psychopathology	26.8 (5.4)	28.2 (5.8)	*t_31,16_* = -0.83, *p*=0.41
BACS^b^	-0.5 (0.9)	-0.5 (0.7)	*t_33,16_ =* 0.10, *p*=0.92
mGAF^c^	42.0 (6.1)	41.1 (7.3)	*t_30,16_ =* 0.46, *p*=0.65
SCoRS^d^	5.1 (2.0)	6.1 (2.0)	*t_31,16_* = -1.57, *p*=0.12
Conversion to psychosis (yes/no) (%)^e^	3/30 (9%)	3/13 (18%)	*χ²* = 0.94, *p*=0.33

All values are shown as means (standard deviations).

ARMS, at-risk mental state; AQ-J, Autism-Spectrum Quotient Japanese version; BACS, Brief Assessment of Cognition in Schizophrenia; H, healthy controls; JART, Japanese Adult Reading Test; mGAF, modified Global Assessment Functioning; PANSS, positive and negative syndrome scale; SCoRS, Schizophrenia Cognition Rating Scale.

a Demographic differences between groups were examined by chi-square or Student’s t-test.

b BACS composite score was calculated by averaging all z-scores of the six primary measures from the BACS.

c Data are ranging from 0 to 100. Healthy subjects generally have a score ranging from 90 to 100.

d Data are ranging from 0 to 10, with larger number representing more worse function.

e Conversion to psychosis was defined according to the psychotic disorder criteria in the Comprehensive Assessment of At-Risk Mental State (Yung et al., 2005).

### 3.2 Comparisons of MMNs between H and ARMS groups

**Figure 1:**
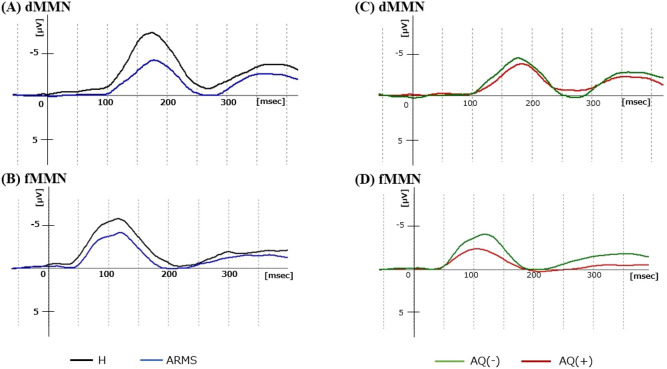
Grand average dMMN and fMMN waveforms at Fz. **(A, B)** show the dMMN and fMMN waveforms of the H (black) and ARMS (blue) groups. **(C, D)** show the dMMN and fMMN waveforms of the AQ(-) (green) and AQ(+) (red) ARMS subgroups. ARMS, at-risk mental state; AQ, Autism -Spectrum Quotient; dMMN, duration mismatch negativity; fMMN, frequency mismatch negativity; H, healthy controls.

As shown in **Table 3A**, there were no statistically significant differences in MMN parameters between the H and ARMS groups. The dMMN amplitude was smaller in ARMS than in H, but this was at trend-level significance [*F*(1,93) = 3.4, *p* = 0.07]. The grand average MMN waveforms are shown in **Figures 1A, B**, with additional detailed scatterplots and waveforms presented in **Supplementary Figures 3, 5**.

**Table 3:** dMMN and fMMN parameters.

A.					
	H	ARMS	Group difference^a^		
	*n=*45	*n=*49	*F*(1, 93)	*p*	*η^2^p*
dMMN amplitude [μV]	5.6 (2.2)	5.0 (2.0)	3.4	0.07^†^	0.036
dMMN latency [msec]	172.9 (19.4)	177.4 (18.6)	0.33	0.57	0.004
fMMN amplitude [μV]	5.8 (2.3)	5.0 (1.8)	0.15	0.70	0.002
fMMN latency [msec]	113.2 (21.0)	115.3 (21.0)	0.38	0.54	0.004

B.					
	AQ(-)	AQ(+)	Group difference^a^		
	*n*=33	*n*=16	*F*(1, 46)	*p*	*η^2^p*
dMMN amplitude [μV]	5.2 (2.1)	4.6 (1.7)	0.38	0.54	0.008
dMMN latency [msec]	178.0 (20.0)	176.1 (17.2)	0.001	0.98	*<* 0.001
fMMN amplitude [μV]	5.1 (2.0)	4.7 (1.6)	0.25	0.62	0.005
fMMN latency [msec]	120.8 (21.8)	104.1 (14.5)	9.8	0.003**	0.18

A. Parameters of H vs ARMS, B. AQ(-) vs AQ(+). Values represent MMN peak amplitudes [μV] and latencies [msec] for each group [mean (SD)].

ARMS, at-risk mental state; AQ, Autism-Spectrum Quotient; dMMN, duration mismatch negativity; fMMN, frequency mismatch negativity; H, healthy controls.

a Differences between groups were examined by ANCOVA with age as a covariate (^†^ *p*<0.1, **p<0.01).

Bold values denote significant differences.

### 3.3 Comparisons of MMNs between AQ(-) and AQ(+) ARMS subgroups

The results are shown in **Table 3B**. The fMMN latency was significantly shorter in AQ(+) than in AQ(-) ARMS subgroups [*F*(1,46) = 9.8, *p* = 0.003, *η^2^p* = 0.18]. This difference remained significant after Bonferroni correction (p<0.0125). There were no significant group differences in other MMN parameters (dMMN amplitude, latency, and fMMN amplitude). To examine the sample size justification, a *post-hoc* power analysis was performed with η²p = 0.18 [fMMN latency, AQ(+) vs. AQ(–)], corresponding to Cohen’s d = 0.94. With group sizes of AQ(+) (n = 16) and AQ(–) (n = 33) and α = 0.05 (two-tailed), the achieved power was 0.85, indicating that the study was adequately powered to detect the observed effect. The grand average MMN waveforms are shown in **Figures 1C, D**, with additional detailed scatterplots and waveforms presented in **Supplementary Figures 4, 6**.

### 3.4 Relationships between MMN parameters and clinical/cognitive indices

**Figure 2:**
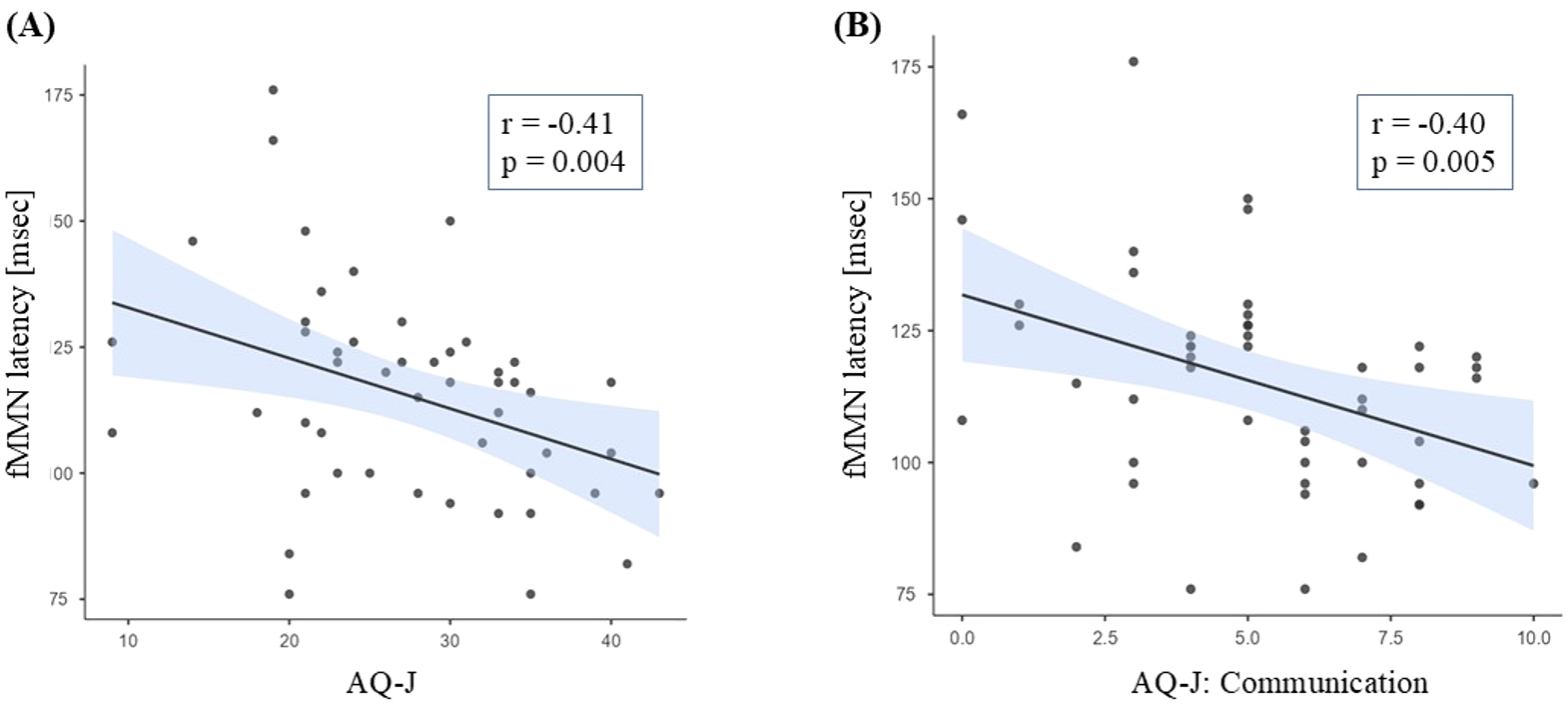
Relationships between fMMN latency and AQ-J total **(A)** or communication subscale **(B)** scores. ARMS, at-risk mental state; AQ-J, Autism-Spectrum Quotient Japanese version; fMMN, frequency mismatch negativity.

The fMMN latency in entire ARMS group was negatively correlated with the AQ-J score (*r*=-0.41, *p*=0.004) (**Figure 2A**). No significant correlations were found between other MMN measures (dMMN amplitude and latency, and fMMN amplitude) and PANSS, BACS, mGAF, or SCoRS scores (**Supplementary Table 7**).

We then investigated the correlations between the fMMN latency and each AQ-J subscale score; the fMMN latency showed a significant negative correlation with the Communication subscale (*r*=-0.40, *p*=0.005) (**Figure 2B**, **Supplementary Table 8**). Imagination was also correlated with fMMN latency, but it did not remain significant after the Bonferroni correction.

## 4 Discussion

To our knowledge, this is the first study demonstrating that fMMN latency is shortened in the ARMS group specifically in individuals who had ASD traits. The fMMN latency was negatively correlated with the AQ-J score in ARMS, suggesting a relationship between the clinical phenotype and underlying neuropsychological mechanisms associated with ASD traits. In previous studies, MMN has been separately studied in ASD and ARMS with only few reports on participants with both ARMS and ASD features. As it is difficult to identify the ASD traits contained in ARMS based on symptoms alone, we believe that the development of biomarkers is important for a more accurate understanding of patient characteristics and for providing with more appropriate support.

In conducting this study, we considered several advantages of using MMN. First, MMN is elicited automatically and is minimally influenced by task demands or antipsychotic exposure, allowing for a direct comparison of neurophysiological processes across subgroups that may differ in clinical status, treatment, or even the ability to comply with task instructions (64). Second, a meta-analysis had shown that individuals with ASD typically exhibit preserved or even shortened MMN latency with relatively intact amplitude, whereas schizophrenia was characterized by marked amplitude reduction and latency prolongation (37, 45, 65). Third, computational models proposed that ASD involves “hyper-precise” predictive coding, accelerating deviance detection, whereas schizophrenia involved hypo-precision and NMDA-receptor dysfunction, damping the same response (61, 66). While NMDA-related dysfunction has been implicated in individuals at risk for psychosis (67), there was no evidence to suggest similar abnormalities in ASD. Based on these considerations, we considered that employing both paradigms would enhance discriminatory power.

To date, as far as we know, only one MMN study has focused on both ASD and psychosis high-risk status; Di Lorenzo et al. (68) compared dMMN and fMMN in youth (9–18 years old) affected by ASD with and without co-occurrent APS (a DSM-5 criteria, and it is nearly equivalent to APS in the CAARMS). They found reduced amplitude particularly for dMMN and somewhat prolonged fMMN latency in the whole ASD group (*n* = 37), but the presence of a concurrent APS condition (*n* = 16) did not affect their MMN findings. However, their results suggested an interaction of ASD and subthreshold psychotic status in showing a robust relationship between higher levels of autistic symptoms and reduced fMMN latency (*r* = -0.81, *p* < 0.001) specifically in the ASD+APS group. Due to differences in strategy and small sample size of subjects with both ASD and high-risk features (*n* = 16 also for this study), it is difficult to directly compare their results with ours; the current study was conducted in the opposite direction (i.e., ARMS cohort as a parent population) to examine MMN features in subjects with overlapping phenotype of ASD and ARMS. Nevertheless, it may be worth noting that both studies suggest a significant role for fMMN latency in the severity of ASD traits, which should be further tested in larger cohorts.

Consistent with previous studies showing reduced dMMN amplitude in various stages of psychosis (i.e., ARMS, FES, and chronic schizophrenia) (29–35), the dMMN amplitude of the entire ARMS group in this study tended to be reduced compared to the H group (**Table 3A**, **Figure 1A**). This finding may reflect the deviation detection disability of the patients in the later part of the temporal time window, which corresponds to the duration of auditory sensory memory in patients with schizophrenia (69, 70). In contrast, as demonstrated in the present (**Table 3A**, **Figure 1B**) and previous (36, 40) studies, the fMMN amplitude does not seem to change in the ARMS group. Similar patterns of reduced dMMN and intact fMMN amplitudes have also been reported in ASD patients (45), implicating that MMN amplitude cannot be useful to distinguish ARMS individuals with ASD traits. Indeed, reduced dMMN amplitude seems to commonly correlate with ASD traits assessed by poor theory of mind in schizophrenia patients, their first-degree relatives, and healthy subjects (71). These findings may also be consistent with a recent study using emotion-related visual task that demonstrated significant association between the interpersonal difficulty, which was commonly indexed as ASD and SSD traits, and MMN amplitude in healthy adults (72).

One major finding of the present study was the shorter fMMN latency in AQ(+) than in AQ(-) ARMS subgroups. Further, the fMMN latency was negatively correlated with the AQ-J score, especially in Communication subscale, in the entire ARMS group. Regarding MMN latency, previous findings have been inconsistent or not well-documented in the SSD or ARMS (41–44). However, it has been hypothesized that MMN indicates the functional state of NMDA (N-methyl-d-aspartate) receptor-mediated neurotransmission, which is associated with the pathophysiology of psychosis (73). NMDA antagonists, such as ketamine and phencyclidine, induce transient schizophrenia-like symptoms in healthy participants and also cause a reduction in dMMN/fMMN amplitude and prolonged fMMN latency (74). Importantly, such prolonged latency is contrary to the finding in ASD, where fMMN latency is shortened at least in certain subtypes (45). Given the role of MMN in predictive coding, where deviant stimulus cause an excessive neural response (75), it is plausible that patients with ASD traits who are characterized by auditory hypersensitivity (76) exhibit a short MMN latency. Taken together, as demonstrated in the present finding, the fMMN latency could help identify underlying ASD traits within the ARMS cohort and guide more tailored interventions. On the other hand, deficiency of communication or interpersonal difficulty, which was associated with shortened fMMN latency in this study, can be a shared ASD and SSD trait phenotype (72). A previous magnetoencephalography study suggested that such phenotype may be associated with dMMN latency ‘delay’ (77). Thus, the role of MMN latency in ASD traits appears to be complex and further research on influencing factors (e.g., stimulation paradigms, demographic and clinical factors) will be required.

To provide a more detailed explanation, within the predictive-coding framework, MMN reflects the brain’s automatic comparison between top-down priors and bottom-up sensory input (78). In schizophrenia and ARMS, reduced dMMN amplitude is thought to be an imprecise index and impaired deviance detection (79), consistent with NMDA-receptor hypofunction and frontotemporal dysconnectivity. Shorter fMMN latency in our AQ(+) subgroup aligns with this “hypo-prior” account: a weaker predictive model would allow deviant tones to breach the threshold for prediction error more rapidly, producing an earlier MMN peak (47, 80). The significant negative correlation between fMMN latency and AQ-Communication further suggests that accelerated prediction-error signaling may underly the social-communication difficulties characteristic of ARMS individuals with ASD traits. Importantly, dMMN amplitude remained blunted across all ARMS participants (although only at a trend level), implying that psychosis-related deviance-detection deficits coexist with ASD-related timing shifts in those who carry both liabilities. These double-dissociated alterations—latency shortening in ASD trait carriers, amplitude reduction in ARMS more broadly—support the notion of a graded neurodevelopmental continuum rather than mutually exclusive pathophysiology. Elucidating such mechanistic heterogeneity is critical for refining early-intervention strategies and for developing MMN-based biomarkers that move beyond diagnosis to personalized stratification.

Although ASD and SSD present distinct clinical features, they may share a common neurobiological mechanism—aberrant prediction error processing (47). Within the predictive coding framework, ASD is associated with weak priors, while schizophrenia is linked to overestimation of prediction errors (80). MMN serves as a neural marker of this process; although it may reflect shared neurophysiological mechanisms, it also has the potential to serve as a valuable tool for differentiation depending on the paradigm employed.

In our cohort, the AQ(+) subgroup exhibited significantly shorter fMMN latency than the AQ(–) subgroup, whereas no group differences were observed in PANSS, BACS, mGAF and SCoRS (see **Table 2**). These findings suggested that the shortened latency was not a marker of general ARMS severity but rather reflected an ASD-linked alteration in pre-attentive sensory prediction. Consistent with this interpretation, fMMN latency correlated negatively with the AQ-Communication subscale (r = –0.46, p = 0.003), while showing no association with PANSS, BACS, mGAF and SCoRS (see **Supplementary Table 7**). Predictive-coding accounts posit that ASD traits are characterized by overly precise sensory priors, leading novel inputs to be processed more rapidly (66); such a mechanism could explain the shortened latency we observed and its specific link to impaired social-communication skills.

This study has some limitations that need to be addressed. First, the sample size was relatively small, limiting the statistical power and generalizability of our results. Second, although the present cohort included more females than males, supplementary analyses indicated that sex had no significant effect on MMN measures and did not influence the main AQ-related findings. These results suggest that the observed effects are unlikely to be attributable to sampling bias. Nonetheless, future studies with more balanced sex ratios are warranted to confirm the generalizability of the findings. Third, eight ARMS patients were taking antipsychotic medication. Two supplementary analyses were performed, and the main results remained unchanged even when the dosage was added as a covariate, and even when patients receiving medication were excluded, the significant difference remained. MMN is less susceptible to the effects of antipsychotics, so it was included in the study, however, for a more rigorous confirmation, it would be desirable to report the results using a cohort consisting only drug-naive participants. Fourth, there were significant group differences in age and premorbid IQ (HC > ARMS), which could influence MMN in both healthy individuals and ARMS. However, there was no difference in age and premorbid IQ between the AQ(-) and AQ(+) groups. Hence, the essential findings of this study are unlikely to have been affected. Fifth, in this study, the ASD traits were assessed using the AQ-J. Although the AQ is a widely used and reliable screening test (48), and validation study was also performed in Japanese version (49), it can only assess the ASD “traits”. For a more accurate assessment, structured tests, for example Autism Diagnostic Observation Schedule, Second Edition (ADOS-2) (81) should be used, and the clinical course, symptoms, and past developmental history should also be rigorously recorded by caregivers. Sixth, because a part of participants was not followed longitudinally to confirm formal diagnoses, the present MMN findings should be viewed only as a screening tool rather than a diagnostic marker. Prospective studies combining MMN with standardized follow-up assessments are warranted to establish diagnostic utility. Seventh, we acknowledge that the classical oddball paradigm does not completely rule out the contribution of stimulus-specific adaptation (SSA). To more rigorously disentangle genuine MMN from N1 adaptation, future studies should consider incorporating paradigms such as the Equiprobable Control Paradigm (82), which better control for refractoriness effects. Eighth, we lacked a reverse-control (counterbalanced) oddball design. We understood this was the most stringent way, however each participant already completed two 1500 trial blocks, and the recording time to include two additional reverse blocks would have substantially increased participant fatigue and artefact contamination, as has been previously reported in long EEG sessions (83). Ninth, our data lacked onset latency data. Peak-latency was retained as our primary result because simulation work indicates that onset-latency shifts were essentially the same extent as peak-latency shifts (84).

In conclusion, our findings support the potential role of MMN as an objective biomarker in clinical settings for early intervention, where the shortening of the fMMN latency in ARMS suggests the possibility of underlying ASD traits. In other words, if there is a shortened fMMN latency in an ARMS case, it could be a trigger for investigating the possibility of ASD traits lurking in the background. As the MMN can be measured easily and noninvasively, our findings may be useful in providing appropriate responses to patients, such as introducing social support tailored to individuals with ASD traits.

## Data Availability

The raw data supporting the conclusions of this article will be made available by the authors, without undue reservation.
