# Supplementary material for: Features of mismatch negativity in an at-risk mental state with the traits associated with the autistic spectrum
Source: Front Psychiatry. 2025 Aug 14;16:1620954. doi: 10.3389/fpsyt.2025.1620954 (PMC12390995; doi:10.3389/fpsyt.2025.1620954)
Supplement: Supplementary file 1 [file DataSheet1.docx]

| Suppl 1. EEG and MMN settings. | | |
| --- | --- | --- |
|  |  |  |
| Sampling rate: 500 Hz | | |
| Reference: Aav | | |
| Bandwidth: 0.53–120 Hz | | |
| Notch filter: 60 Hz | | |
| Sound stimulus delivery method: through binaural ears, through headphones | | |
| Distraction: silent animation | | |
| Sound stimulus (through headphones) | |  |
|  | dMMN block | fMMN block |
| No.trials (Standard/Deviant) | 1350 / 150 | 1350 / 150 |
| Standard tone | 1000 Hz, 50 ms | 1000 Hz, 50 ms |
| Deviant tone | 1000 Hz, 100 ms | 1500 Hz, 50 ms |
| Probability (Deviant) | 10% | 10% |
| ISI / SOA | 500 ms / 550 ms (standard) or 600 ms (deviant) | 500 ms / 550 ms |
| Rise/fall time | 10 ms | 10 ms |
| Sound level | 60 dB SPL (binaural) | 60 dB SPL (binaural) |
|  |  |  |
| Abbreviations: EEG, electroencephalography; dMMN, duration mismatch negativity; fMMN, frequency mismatch negativity; ISI, inter-stimulus interval; SOA, stimulus-onset asynchrony; SPL, sound pressure level. | | |

| Suppl 2. Detailed information on the AQ-J subscales. | | | | | | | | | | | | | | | | | | |  |
| --- | --- | --- | --- | --- | --- | --- | --- | --- | --- | --- | --- | --- | --- | --- | --- | --- | --- | --- | --- |
|  |  |  |  |  |  |  |  |  |  |  |  |  |  |  |  |  |  |  |  |
|  |  | H | | | ARMS | | | | | |  | Group difference^a^ | | |  | Post-hoc analysis^a^ | | |  |
|  |  |  |  |  | AQ(-) | | | AQ(+) | | |  |  |  |  |  |  |  |  |  |
|  |  | *n=*45 | | | *n*=33 | | | *n*=16 | | |  | *F* | *p* | *η^2^* |  | AQ(-) vs AQ(+) | AQ(-) vs H | AQ(+) vs H |  |
|  |  |  |  |  |  |  |  |  |  |  |  |  |  |  |  |  |  |  |  |
| Social interaction | | 3.6 (2.5) | | | 5.2 (2.6) | | | 8.4 (1.7) | | |  | 23.1 | **<0.001**** | 0.34 |  | ***t=*-4.1*, p*<0.001**** | ***t=*2.9*, p=*0.014*** | ***t=*6.8*, p<*0.001**** |  |
| Tolerance for change | | 4.0 (1.8) | | | 5.5 (2.2) | | | 8.3 (1.3) | | |  | 31.2 | **<0.001**** | 0.41 |  | ***t*=-5.0*, p*<0.001**** | ***t=*3.4*, p=*0.003**** | ***t=*7.8*, p<*0.001**** |  |
| Attention to detail | | 4.4 (2.1) | | | 5.2 (2.5) | | | 6.8 (2.1) | | |  | 6.6 | **0.002**** | 0.13 |  | *t*=-2.3, *p*=0.07 | *t=*1.5*, p=*0.39 | ***t=*3.6*, p=*0.002**** |  |
| Communication patterns | | 3.0 (1.8) | | | 3.8 (1.9) | | | 7.7 (1.4) | | |  | 41.7 | **<0.001**** | 0.48 |  | ***t*=-6.4, *p<*0.001**** | *t=*2.1*, p=*0.13 | ***t=*9.1*, p<*0.001**** |  |
| Imagination | | 2.9 (1.6) | | | 3.5 (1.7) | | | 5.1 (1.6) | | |  | 10.2 | **<0.001**** | 0.18 |  | ***t=-3.1, p=*0.008****** | *t=*1.5*, p=*0.39 | ***t=*4.5*, p<*0.001**** |  |
| AQ-J total score | | 18.0 (5.7) | | | 23.2 (5.7) | | | 36.2 (3.3) | | |  | 67.2 | **<0.001**** | 0.60 |  | ***t*=-7.3, *p*<0.001**** | ***t=*4.3*, p<*0.001**** | ***t=*11.6*, p<*0.001**** |  |
|  |  |  |  |  |  |  |  |  |  |  |  |  |  |  |  |  |  |  |  |
| Values represent AQ-J subscale scores for each group [mean (SD)].  Abbreviations: ARMS, at-risk mental state; AQ-J, Autism-Spectrum Quotient Japanese version; AQ(–), group with AQ scores below threshold; AQ(+), group with AQ scores above threshold; H, healthy controls. ^a^Differences between groups were examined by ANOVA with post-hoc Bonferroni correction (**p*<0.05, ***p*<0.01). | | | | | | | | | | | | | | | | | | |  |

Suppl 3. Scatterplots of MMN parameters.


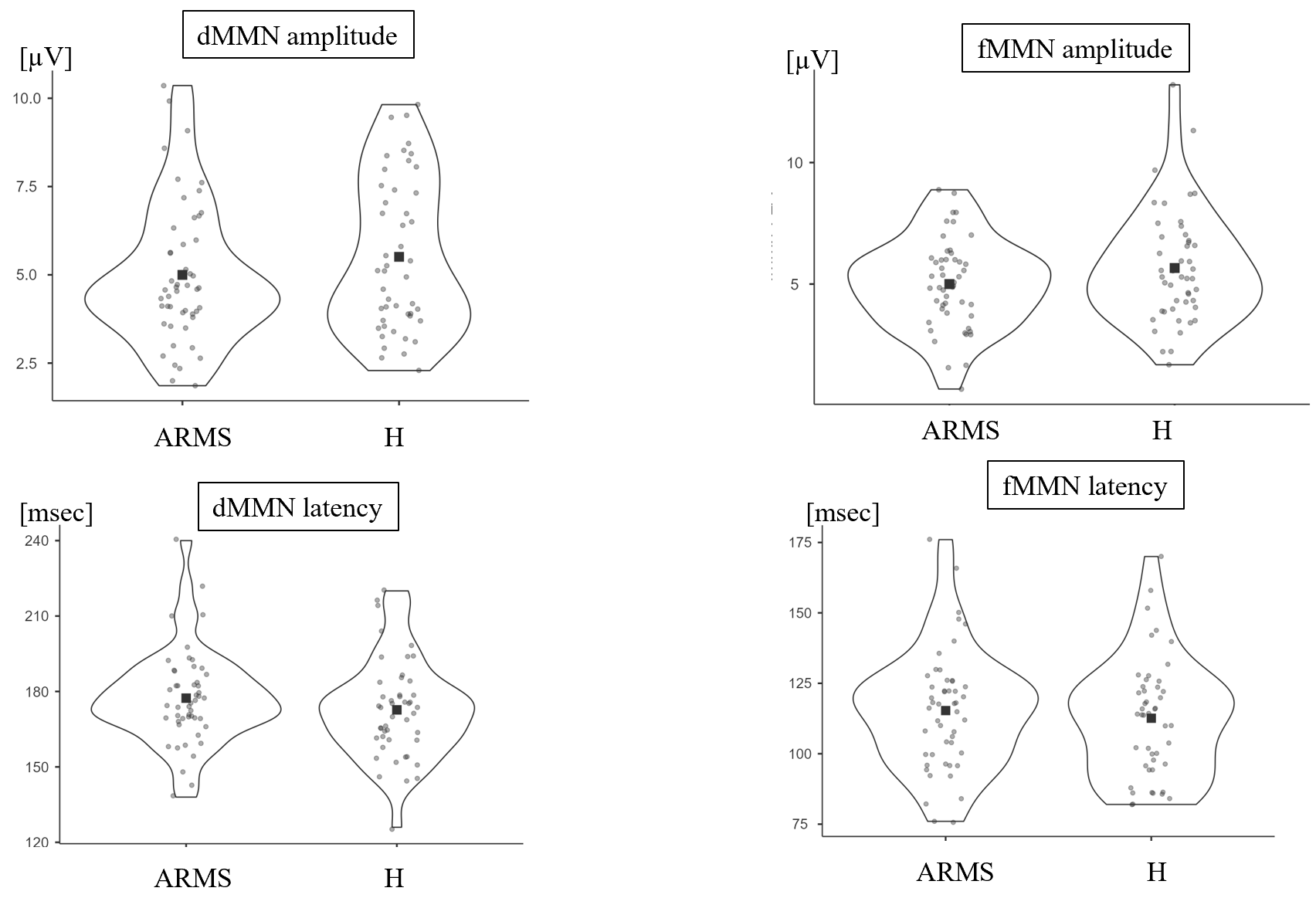


Abbreviations: ARMS, at-risk mental state; dMMN, duration mismatch negativity; fMMN, frequency mismatch negativity; H, healthy controls.

Suppl 4. Scatterplots of MMN parameters.


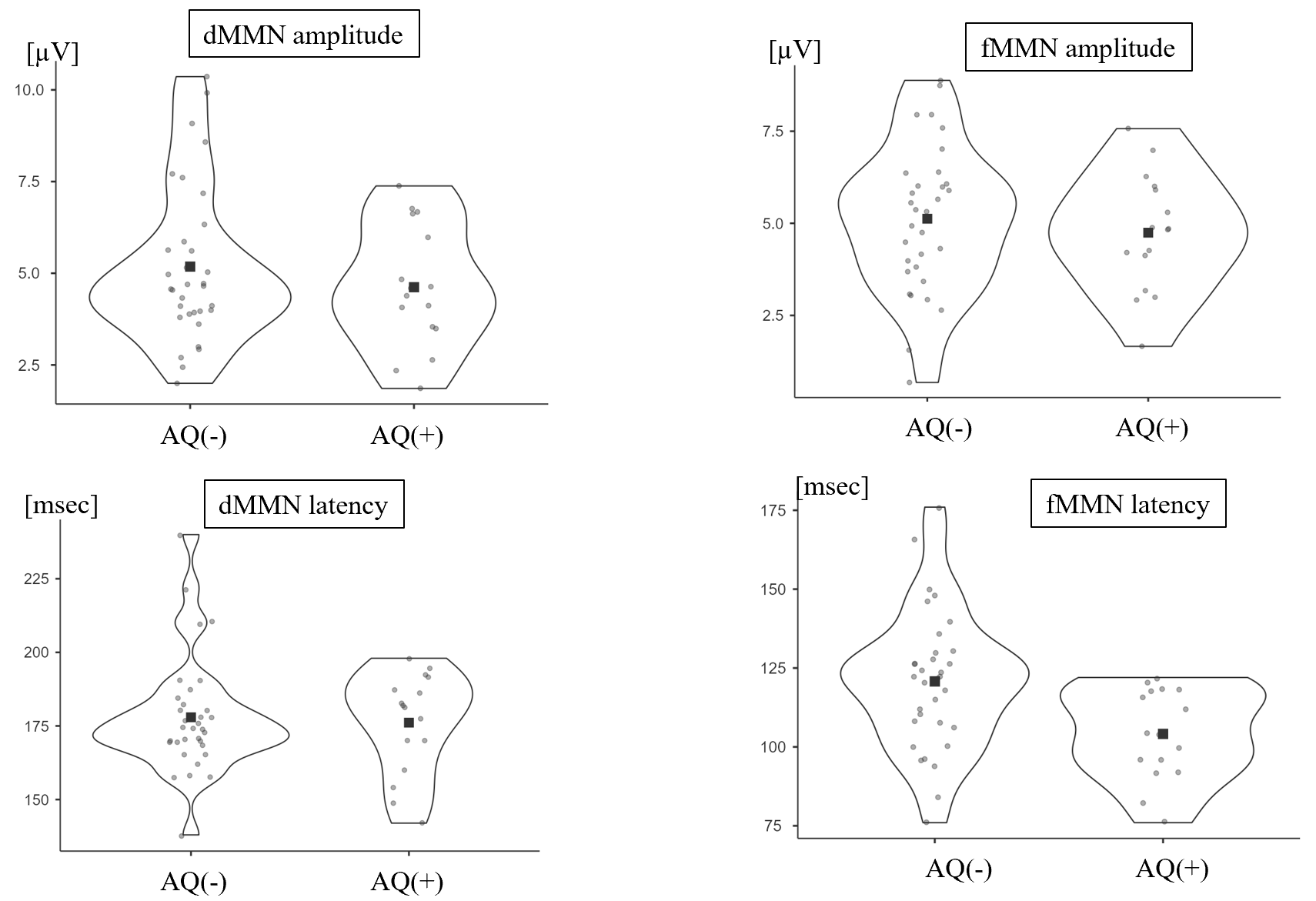


Abbreviations: AQ(+); Autism-Spectrum Quotient Japanese version; AQ(–), group with AQ scores below threshold; AQ(+), group with AQ scores above threshold; dMMN, duration mismatch negativity; fMMN, frequency mismatch negativity.

Suppl 5. Grandaverage waveforms of dMMN and fMMN at Fz in ARMS and H (details).


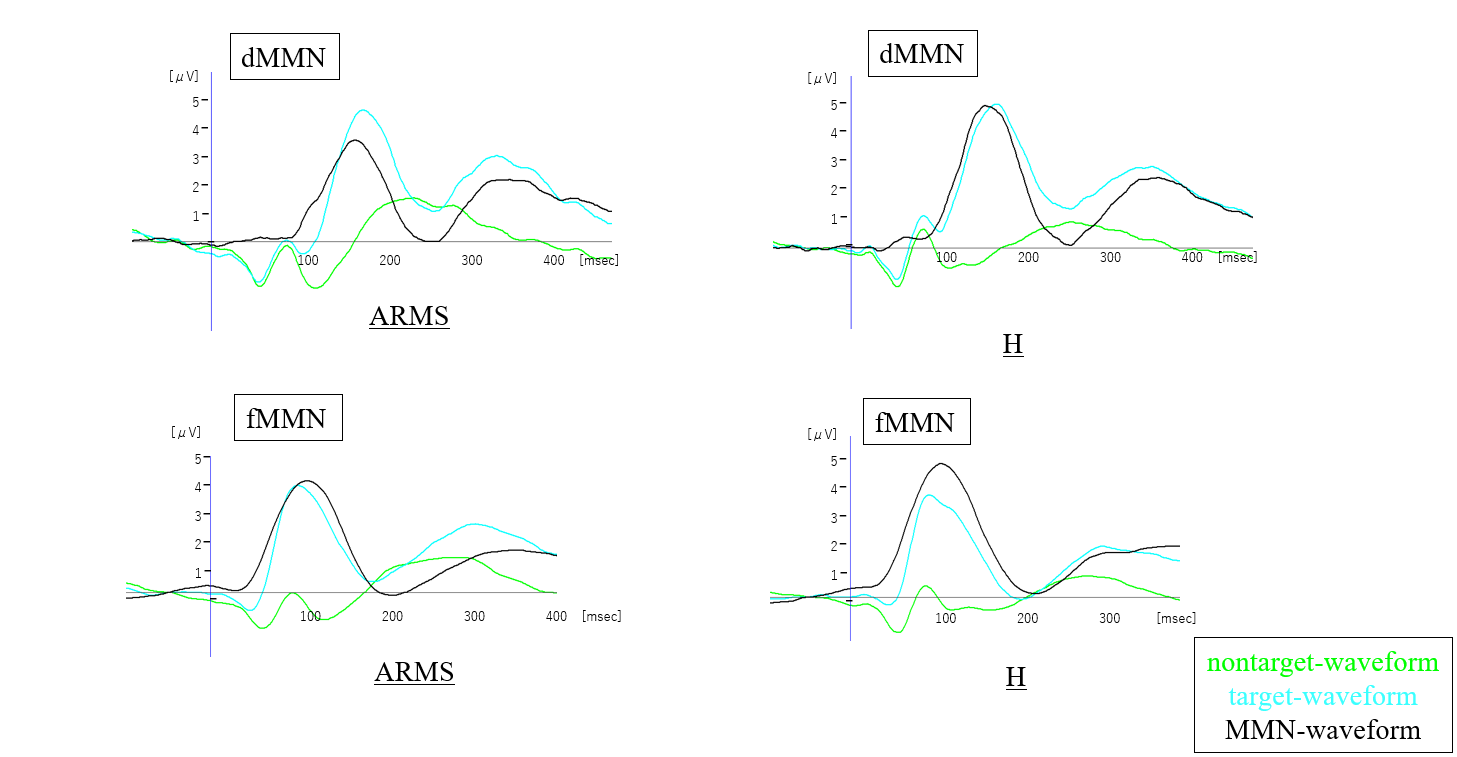


Nontarget waveforms, target waveforms, and MMN waveforms are shown in light green, light blue, and black, respectively.

Abbreviations: ARMS, at-risk mental state; dMMN, duration mismatch negativity; fMMN, frequency mismatch negativity; H, healthy controls.

Suppl 6. Grandaverage waveforms of dMMN and fMMN at Fz in AQ(+) and AQ(-) (details, original ERPs).


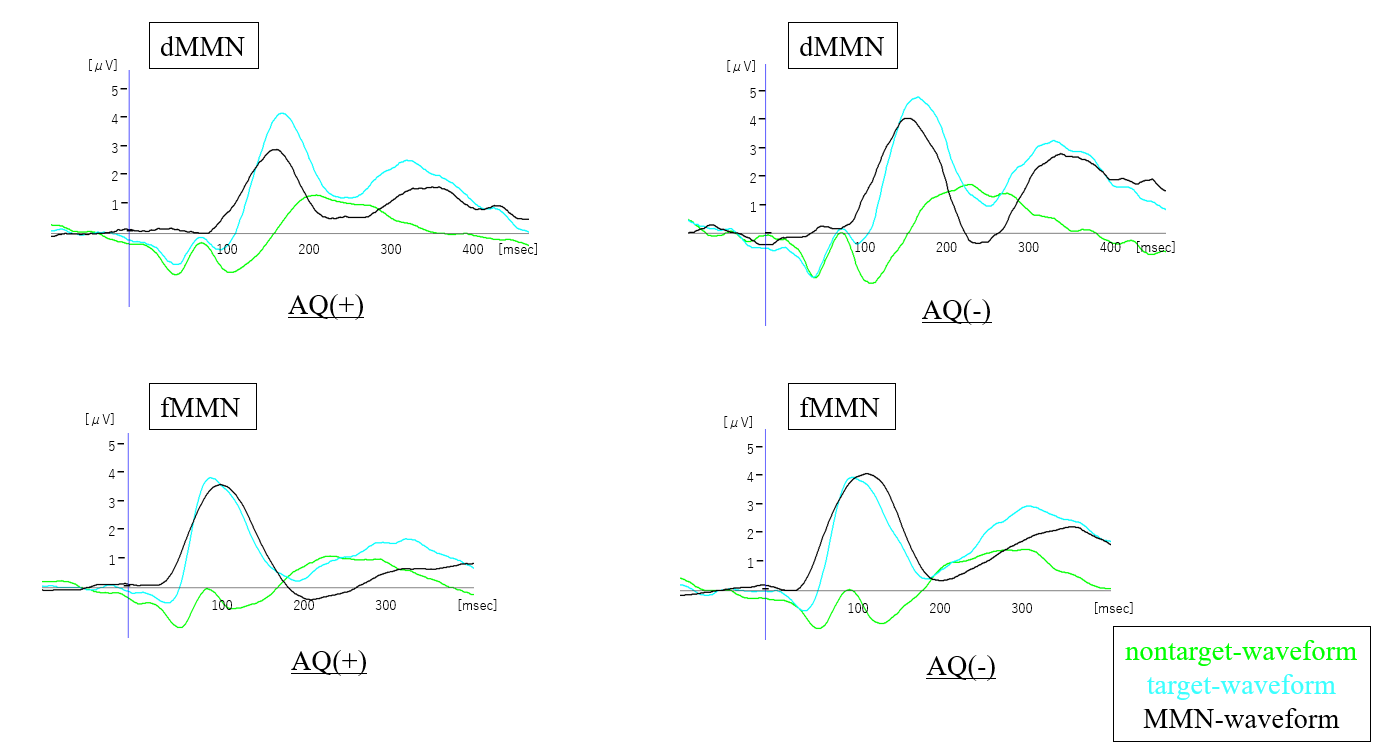


Nontarget waveforms, target waveforms, and MMN waveforms are shown in light green, light blue, and black, respectively.

Abbreviations: AQ(–), group with AQ scores below threshold; AQ(+), group with AQ scores above threshold; dMMN, duration mismatch negativity; fMMN, frequency mismatch negativity.

| Suppl 7. Relationships between dMMN/fMMN parameters and clinical/cognitive indices in ARMS. | | | | | | | | | | |
| --- | --- | --- | --- | --- | --- | --- | --- | --- | --- | --- |
|  |  |  |  |  |  |  |  |  |  |  |
| Table5 | PANSS | | BACS | | mGAF | | SCoRS | | AQ-J | |
|  | *r* | *p* | *r* | *p* | *r* | *p* | *r* | *p* | *r* | *p* |
| dMMN amplitude | -0.02 | 0.89 | 0.01 | 0.93 | 0.18 | 0.25 | 0.14 | 0.37 | -0.003 | 0.99 |
| dMMN latency | 0.21 | 0.17 | -0.04 | 0.79 | -0.17 | 0.26 | -0.12 | 0.43 | 0.14 | 0.33 |
| fMMN amplitude | 0.16 | 0.28 | -0.23 | 0.11 | -0.06 | 0.69 | -0.01 | 0.96 | -0.01 | 0.93 |
| fMMN latency | 0.22 | 0.14 | -0.27 | 0.07 | -0.02 | 0.91 | -0.02 | 0.88 | -0.41 | **0.004**** |
|  |  |  |  |  |  |  |  |  |  |  |
| Values are calculated by Pearson’s correlation coefficient using semi-partial correlation analysis, where only MMN parameters are controlled by age as a covariate (***p*<0.01).  Abbreviations: AQ-J, Autism-Spectrum Quotient Japanese version; BACS, Brief Assessment of Cognition in Schizophrenia; dMMN, duration mismatch negativity; fMMN, frequency mismatch negativity; mGAF, modified Global Assessment Functioning; PANSS, positive and negative syndrome scale; SCoRS, Schizophrenia Cognition Rating Scale. | | | | | | | | | | |

| Suppl 8. Relationships between fMMN latency and AQ-J subscores. | | | | | | | |  |  |  |
| --- | --- | --- | --- | --- | --- | --- | --- | --- | --- | --- |
|  |  |  |  |  |  |  |  |  |  |  |
|  | Social skill | | Attention switching | | Attention to detail | | Communication | | Imagination | |
|  | *r* | *p* | *r* | *p* | *r* | *p* | *r* | *p* | *r* | *p* |
| fMMN latency | -0.21 | 0.15 | -0.23 | 0.13 | -0.22 | 0.13 | -0.40 | **0.005**** | -0.29 | **0.04*** |
|  |  |  |  |  |  |  |  |  |  |  |
| Values are calculated by Pearson’s correlation coefficient using semi-partial correlation analysis, where only fMMN parameters are controlled by age as a covariate (**p*<0.05, ***p*<0.01). Abbreviations: AQ-J, Autism-Spectrum Quotient Japanese version; fMMN, frequency mismatch negativity. | | | | | | | | | | |
